# Prognostic Value of the Blood Urea Nitrogen-to-Albumin Ratio (BAR) for Long-Term Survival Outcomes Among ST-Segment Elevation Myocardial Infarction Patients Undergoing Primary Percutaneous Coronary Intervention

**DOI:** 10.31083/RCM48910

**Published:** 2026-06-26

**Authors:** Fazhi Yang, Ningli Zhang, Jie Zou, Hongxia Li, Ping Xia, Yanqing Liu, Xinuo Ma, Yujuan Peng, Yunyun Xu, Lixing Chen

**Affiliations:** ^1^Department of Cardiology, Kunming Medical University First Affiliated Hospital, 650032 Kunming, Yunnan, China; ^2^Department of Anesthesiology, Kunming Medical University Second Affiliated Hospital, 650101 Kunming, Yunnan, China; ^3^Department of Emergency Medicine, Kunming Medical University First Affiliated Hospital, 650032 Kunming, Yunnan, China; ^4^Department of Cardiology, YAN’AN Hospital of Kunming City, 650051 Kunming, Yunnan, China

**Keywords:** blood urea nitrogen, albumin, ST-elevation myocardial infarction, percutaneous coronary intervention

## Abstract

**Background:**

The blood urea nitrogen-to-albumin ratio (BAR) has emerged as an independent prognostic marker of mortality in acute myocardial infarction (AMI). This study aimed to clarify the association between BAR and long-term survival outcomes in patients with ST-segment elevation myocardial infarction (STEMI) undergoing primary percutaneous coronary intervention (PPCI).

**Methods:**

We retrospectively analyzed 1099 consecutive STEMI patients who underwent PPCI at the First Affiliated Hospital of Kunming Medical University between June 2018 and January 2023. Kaplan–Meier survival analysis and restricted cubic splines (RCS) were used to compare mortality among patients. Cox regression and subgroup analyses were performed to assess the associations between the BAR and the prespecified endpoints. The predictive efficacy of the BAR was quantified using receiver operating characteristic (ROC) curve analysis.

**Results:**

Patients with higher and elevated BARs had exhibited significantly worse long-term outcomes. After adjusting for confounders, the BAR remained an independent predictor of all-cause mortality (hazard ratio (HR) = 1.118, 95% confidence interval (CI): 1.049–1.193, *p* = 0.001) and cardiovascular mortality (HR = 1.107, 95% CI: 1.035–1.184, *p* = 0.003). The areas under the ROC (AUROC) curve for the BAR was 0.720 (95% CI: 0.693–0.747) for all-cause mortality and 0.721 (95% CI: 0.694–0.747) for cardiovascular mortality.

**Conclusions:**

The BAR is an independent prognostic biomarker for long-term all-cause and cardiovascular mortality in STEMI patients post-PPCI, highlighting the potential clinical utility of this ratio for in risk stratification.

## 1. Introduction

Acute coronary syndrome (ACS) encompasses a spectrum of serious ischemic syndromes caused by altered coronary blood flow, and it remains a leading contributor to the worldwide burden of cardiovascular illness and death. According to data from the Organization for Economic Cooperation and Development (OECD), patients with an acute myocardial infarction (AMI) or ischemic stroke (IS) together represent roughly 85% of all cardiovascular and cerebrovascular disease cases, indicating the significant severity of this disease [1]. Among its heterogeneous presentations, ST-segment elevation myocardial infarction (STEMI) and non-ST-segment elevation myocardial infarction (NSTEMI) represent the most frequently encountered and clinically consequential variants, with STEMI distinguished by its particularly aggressive natural history. This subtype manifests abruptly, evolves rapidly, and carries an alarmingly elevated fatality rate, positioning it as a persistent and formidable challenge in contemporary cardiovascular medicine. The clinical diagnosis of a STEMI relies on three core features: prolonged anginal symptoms, documented myocardial ischemia, and typical electrocardiographic changes, which together form its diagnostic framework [2].

Recent clinical evidence indicates that both short- and long-term mortality following STEMI have declined substantially, largely attributable to the widespread adoption of percutaneous coronary intervention (PCI) and advances in pharmacological therapy. Nevertheless, approximately 3%–5% of patients still die within 30 days of diagnosis, and this proportion increases to 7%–8% within the first year [3]. Therefore, the early identification of patients with favorable prognostic profiles and the development of reliable risk-stratification models are of considerable clinical importance, as they may facilitate the timely implementation of individualized treatment strategies and targeted risk-factor interventions, ultimately improving both short-term outcomes and long-term prognosis.

Blood urea nitrogen (BUN), the principal end metabolite of human protein catabolism, is primarily eliminated by renal excretion [4]. Recognized as a marker of renal function, BUN has been linked to outcomes in diverse conditions, such as acute pancreatitis, chronic obstructive pulmonary disease, and acute ischemic stroke [5,6,7]. In recent years, its potential in predicting cardiovascular events has received increasing interest. Jujo et al. [8] reported that persistently elevated in-hospital BUN levels correlate with a higher risk of cardiovascular mortality and rehospitalization in patients with heart failure.

Albumin (ALB), the most prevalent circulating protein in plasma, plays critical roles in binding and transporting diverse endogenous and exogenous molecules, regulating plasma oncotic pressure, and modulating circulatory system physiology [9]. Currently, it is well established that ALB serves as a marker of both systemic inflammation and nutritional status. Furthermore, ALB is intricately involved in antioxidant, anticoagulant, and antiplatelet activities [10,11].

The blood urea nitrogen-to-albumin ratio (BAR), derived from BUN and ALB, has recently emerged as a promising prognostic biomarker for multiple conditions, including heart failure, pulmonary embolism, and lung cancer [12,13,14]. Several studies have further validated its role as an independent risk factor for mortality in patients with AMI [15,16]. Despite these advances, the association between BAR and long-term outcomes in patients with STEMI undergoing primary percutaneous coronary intervention (PPCI) remains unexplored. The aim of this study is to evaluate the predictive value of BAR for the long-term prognosis of STEMI patients undergoing PPCI.

## 2. Materials and Methods

### 2.1 Study Population

We reviewed the medical records of 1341 consecutive patients diagnosed with STEMI who underwent PPCI between June 2018 and January 2023 at the First Affiliated Hospital of Kunming Medical University. Acute STEMI was defined according to the fourth universal definition of myocardial infarction: (1) typical chest discomfort persisting for >30 minutes; (2) new ST-segment elevation at the J point ≥2 mm (0.2 mV) in males or ≥1.5 mm (0.15 mV) in females across at least two contiguous electrocardiogram (ECG) leads on admission; and (3) elevated levels of cardiac biomarkers [17].

**Fig. 1.**
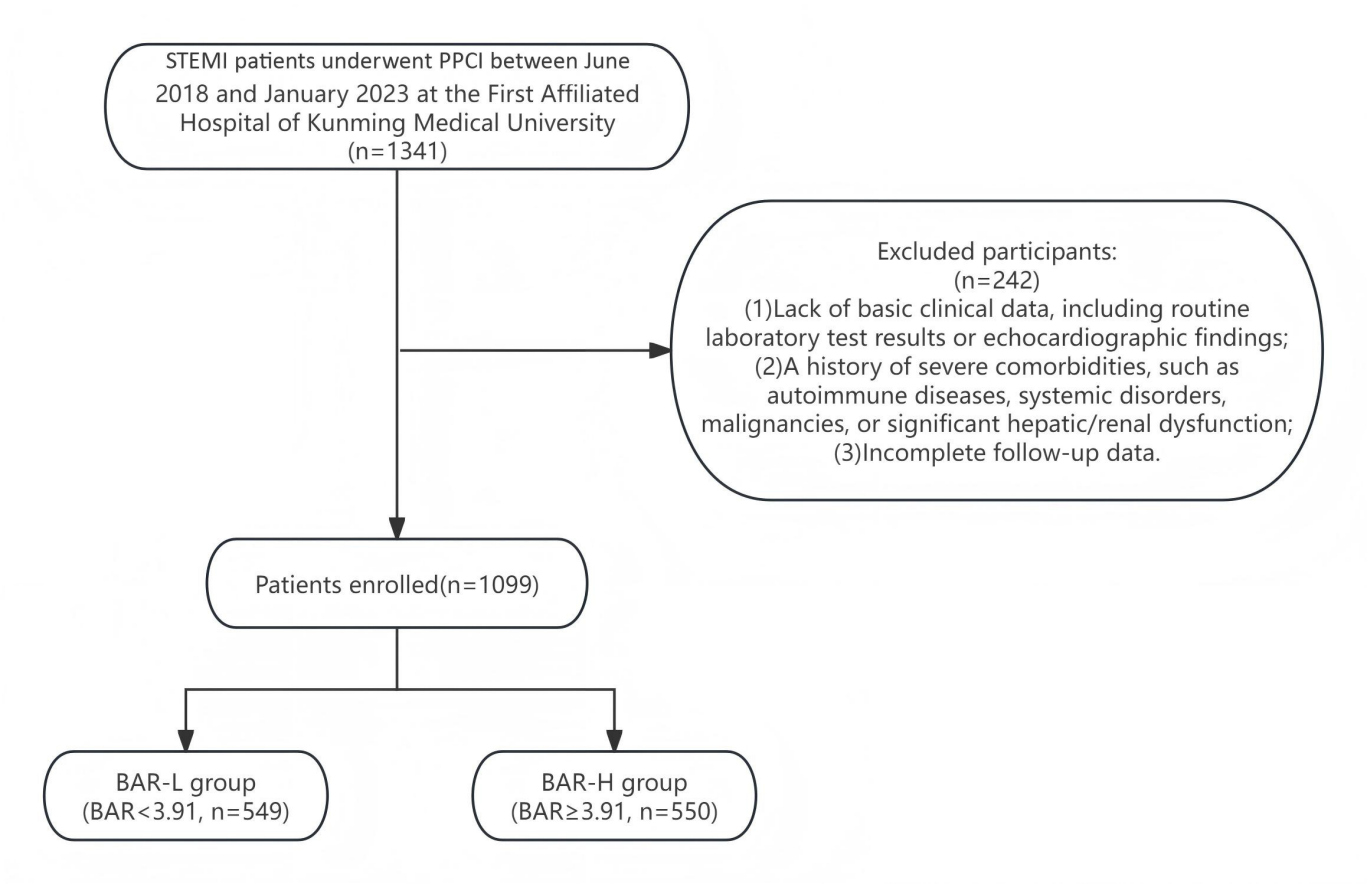
**Flow chart of patient selection for this cohort study**. STEMI, ST-segment elevation myocardial infarction; PPCI, primary percutaneous coronary intervention; BAR, blood urea nitrogen-to-albumin ratio.

Individuals were omitted from the study if they fulfilled any of the following exclusion criteria: (1) lack of basic clinical data, including routine laboratory test results or echocardiographic findings; (2) a history of severe comorbidities, such as autoimmune diseases, systemic disorders, malignancies, or significant hepatic/renal dysfunction; (3) incomplete follow-up data. A total of 1099 patients were retained for final analysis following the application of the exclusion criteria (Fig. 1).

### 2.2 Data Collection

This study collected patient data from the Electronic Medical Record (EMR) system at the First Affiliated Hospital of Kunming Medical University, systematically extracting detailed medical history, complete medication records, demographic characteristics, baseline clinical features, hematological and biochemical parameters, as well as echocardiographic measurements. Blood samples had been taken before any therapeutic measurements, including routine blood tests, myoglobin, creatine kinase isoenzyme MB (CK-MB), and cardiac troponin-I (cTnI). After fasting for 12 hours, other blood samples were collected in strict accordance with standard procedures and were sent to the laboratory of the First Affiliated Hospital of Kunming Medical University for immediate testing according to standard techniques. The detection indicators included sodium, potassium, ALB, alanine aminotransferase (ALT), aspartate aminotransferase (AST), serum total bilirubin (STB), conjugated bilirubin (CB), unconjugated bilirubin (UCB), BUN, creatinine, uric acid (UA), fasting blood glucose (FBG), total cholesterol (TC), triglycerides (TG), high-density lipoprotein cholesterol (HDL-C), and low-density lipoprotein cholesterol (LDL-C). The BAR was calculated by dividing BUN (mmol/L) by ALB (g/L). The Gensini score was calculated based on a guideline developed by Rampidis et al. [18] in 2019. In addition to these clinical data, we documented coronary angiographic findings obtained in the cardiac catheterization laboratory of the First Affiliated Hospital of Kunming Medical University, including the extent, location, degree of stenosis, site and quantity of stent implantation, and intraoperative Thrombolysis in Myocardial Infarction (TIMI) grading.

### 2.3 Coronary Angiography and Primary Percutaneous Coronary Intervention

After admission, STEMI patients followed a standard treatment plan and underwent coronary angiography (CA) by experienced senior cardiologists. For newly diagnosed STEMI patients, the first loading dose (aspirin plus P2Y12 inhibitor) was administered as early as possible before PPCI. In our cohort, a small proportion of STEMI patients did not receive aspirin and/or a P2Y12 inhibitor due to the following reasons: (1) documented absolute contraindications such as active major bleeding; (2) pre-hospital administration already completed before hospital arrival, and thus not recorded as a separate in-hospital dose. Data were extracted from the hospital information system (HIS; Neusoft Corporation, Shenyang, Liaoning, China); (3) missing documentation in electronic health records for a few cases due to transfer from external facilities.

CA and PPCI procedures were conducted in the cardiac catheterization laboratory, with documentation of lesion characteristics including anatomical location, extent, and morphology, as well as quantitative assessments of the severity of the stenosis through both visual estimation and quantitative coronary angiography where available. Procedural details such as the number and type of stents deployed, stent positioning, and use of adjunctive therapies were recorded, along with intraoperative TIMI flow grades. TIMI flow grade during the procedure was assessed in real time by the interventional cardiologist performing the PPCI, based on visual estimation of coronary artery opacification and clearance of contrast material, following standard TIMI flow criteria. To ensure data accuracy and minimize observer bias, all angiographic measurements and interpretations were independently reviewed and validated by two experienced interventional cardiologists, with any discrepancies resolved through consensus discussion. Successful complete revascularization refers to successful stent implantation, intraoperative residual stenosis <20%, and intraoperative TIMI flow reaching grade III.

### 2.4 Clinical Endpoint

The study evaluated two primary endpoints: all-cause mortality and cardiovascular mortality. Cardiovascular mortality was specifically defined as death resulting from decompensated heart failure, life-threatening arrhythmias (sustained ventricular tachycardia or ventricular fibrillation), acute myocardial infarction, aortic dissection, peripheral arterial disease complications, or sudden cardiac death. Survival outcomes were collected by structured telephone interviews.

### 2.5 Statistical Analysis

Statistical analyses were conducted using SPSS version 26.0 (IBM Corp., Armonk, NY, USA) and R version 4.3.2 (R Foundation for Statistical Computing, Vienna, Austria). Patients were stratified into two groups by the median BAR value: BAR-L group (BAR <3.91, n = 549) and BAR-H group (BAR ≥3.91, n = 550). Continuous baseline variables are summarized as median with interquartile range (IQR), while categorical variables are presented as counts and proportions. For comparisons between BAR-L and BAR-H groups: normally distributed data were analyzed via an independent samples *t*-test, non-normally distributed data via the Mann-Whitney U test, and categorical data via the χ^2^ test. Survival probabilities over time were visualized using Kaplan–Meier curves, and differences between groups were tested via the log-rank test. To investigate the association between BAR and all-cause/cardiovascular mortality in STEMI patients after PPCI, univariate and multivariate Cox proportional hazards models were fitted, adjusted for confounding factors. The results were reported as hazard ratios (HRs) with 95% confidence intervals (CIs). Restricted cubic splines (RCS) were applied to explore potential nonlinear dose–response relationships between BAR and mortality outcomes. Subgroup analyses were conducted to examine how BAR correlates with all-cause mortality and cardiovascular mortality across distinct patient subgroups. Receiver operating characteristic (ROC) curves were generated for BUN, ALB, and BAR to assess their predictive accuracy for long-term survival outcomes, with AUC comparisons made using the Delong's test. A *p*-value of less than 0.05 was deemed statistically significant for every analysis.

## 3. Results

### 3.1 Baseline Characteristics

Following exclusions for loss to follow-up or missing data, 1099 STEMI patients who underwent PPCI were retained for final analysis—549 in the BAR-L group (BAR <3.91) and 550 in the BAR-H group (BAR ≥3.91). The baseline characteristics, medical history, laboratory data, and medication are presented in Table 1. In this study, the average age of the population was 60.74 years, with 84.4% of patients being male. Compared with the BAR-L group, patients in the BAR-H group were older and had significantly higher levels of monocytes (MO), C-reactive protein (CRP), BUN, creatinine, UA, and serum sodium, as well as a higher Gensini score. Conversely, they had significantly lower body mass index (BMI), systolic blood pressure (SBP), diastolic blood pressure (DBP), red blood cell (RBC) count, hemoglobin (HB) level, lymphocyte (LYM) count, platelet (PLT) count, serum albumin (ALB), estimated glomerular filtration rate (eGFR), potassium level, and left ventricular ejection fraction (LVEF). In terms of comorbidities, patients in the BAR-H group had a higher prevalence of hypertension, diabetes mellitus, atrial fibrillation, and heart failure. In contrast, the prevalence of hyperlipidemia was higher in the BAR-L group (*p* < 0.05).

**Table 1.** **Baseline characteristics according to BAR**.

Variables		Total population	BAR-L (BAR <3.91)	BAR-H (BAR ≥3.91)	*p* value
		n = 1099	n = 549	n = 550	
Basic characteristics					
Age, years		60.74 ± 12.07	57.33 ± 11.95	64.14 ± 11.21	<0.001
Sex, female, %		171 (15.6)	80 (14.6)	91 (16.5)	0.367
BMI, kg/m^2^		24.29 ± 3.13	24.55 ± 3.13	24.03 ± 3.11	0.006
SBP, mmHg		126.68 ± 23.59	129.60 ± 22.58	123.76 ± 24.22	<0.001
DBP, mmHg		81.20 ± 16.12	83.87 ± 15.48	78.54 ± 16.32	<0.001
HR, beat/minute		78.85 ± 17.07	79.71 ± 16.23	78.00 ± 17.83	0.097
Smoking, %		627 (57.1)	329 (59.9)	298 (54.2)	0.054
Medical history					
Hypertension, %		615 (56.0)	281 (51.2)	334 (60.7)	0.001
Diabetes, %		346 (31.5)	156 (28.4)	190 (34.5)	0.029
Hyperlipidemia, %		350 (31.8)	198 (36.1)	152 (27.6)	0.003
Atrial fibrillation, %		25 (2.3)	5 (0.9)	20 (3.6)	0.002
Heart failure, %		299 (27.2)	109 (19.9)	190 (34.5)	<0.001
Laboratory data					
RBC, 10^12^/L		4.91 ± 0.66	5.05 ± 0.66	4.77 ± 0.70	<0.001
Hemoglobin, g/L		152.84 ± 23.01	157.25 ± 24.30	148.40 ± 20.74	<0.001
WBC, 10^9^/L		11.03 ± 4.59	11.20 ± 5.23	10.87 ± 3.83	0.238
NEU, 10^9^/L		8.50 ± 3.74	8.46 ± 3.50	8.54 ± 3.97	0.719
LYM, 10^9^/L		1.70 ± 0.83	1.80 ± 0.91	1.61 ± 0.74	<0.001
MO, 10^9^/L		0.65 ± 0.35	0.62 ± 0.33	0.67 ± 0.38	0.030
PLT, 10^9^/L		225.32 ± 72.77	230.15 ± 76.15	220.45 ± 68.92	0.028
CRP, mg/L		3.38 (1.20, 12.80)	3.05 (1.00, 9.01)	4.96 (1.47, 22.65)	<0.001
Albumin, g/L		39.63 ± 4.48	41.18 ± 3.94	38.09 ± 4.45	<0.001
ALT, IU/L		43.00 (29.00, 63.00)	43.00 (29.00, 63.00)	42.50 (29.50, 63.25)	0.997
AST, IU/L		73.50 (32.00, 175.85)	72.00 (31.25, 176.85)	74.90 (32.00, 174.60)	0.743
STB, µmol/L		12.00 (8.80, 17.20)	12.20 (9.10, 17.30)	11.90 (8.48, 17.10)	0.323
CB, µmol/L		3.60 (2.50, 5.40)	3.50 (2.50, 5.20)	3.60 (2.40, 5.50)	0.477
BUN, mmol/L		5.60 (4.53, 6.90)	4.54 (3.84, 5.07)	6.90 (6.13, 8.22)	<0.001
Cre, µmol/L		89.70 (76.40, 103.80)	83.90 (72.10, 95.10)	95.25 (81.28, 114.35)	<0.001
UA, µmol/L		384.55 (317.75, 465.98)	368.40 (306.90, 448.70)	405.40 (327.00, 485.70)	<0.001
eGFR, mL/min/1.73 m^2^		72.28 (55.55, 90.95)	81.94 (66.35, 99.86)	61.31 (46.93, 79.11)	<0.001
Potassium, mmol/L		3.73 ± 0.54	3.68 ± 0.51	3.77 ± 0.56	0.003
Sodium, mmol/L		138.89 ± 3.73	139.19 ± 3.05	138.59 ± 4.25	0.007
TC, mmol/L		4.44 (3.70, 5.17)	4.54 (3.84, 5.33)	4.35 (3.57, 5.03)	<0.001
TG, mmol/L		1.48 (1.04, 2.06)	1.52 (1.09, 2.16)	1.44 (1.01, 1.97)	0.015
HDL-C, mmol/L		1.04 (0.88, 1.22)	1.04 (0.88, 1.22)	1.04 (0.88, 1.22)	0.839
LDL-C, mmol/L		2.80 (2.19, 3.48)	2.94 (2.32, 3.62)	2.70 (2.03, 3.34)	<0.001
Myoglobin, ng/mL		156.35 (58.45, 367.73)	148.70 (53.52, 334.88)	160.46 (61.96, 397.59)	0.112
Cardiac Troponin I, ng/mL		2.37 (0.12, 15.25)	1.81 (0.10, 13.13)	3.02 (0.15, 17.79)	0.077
CK-MB, ng/mL		19.60 (3.58, 72.10)	24.00 (3.74, 74.24)	15.80 (3.45, 68.14)	0.344
BAR		3.91 (3.13, 5.03)	3.13 (2.65, 3.50)	5.03 (4.42, 6.13)	<0.001
Echocardiographic data					
LVEF, %		61.36 ± 11.55	63.34 ± 10.84	59.39 ± 11.90	<0.001
LVFS, %		34.01 ± 8.44	35.25 ± 8.19	32.77 ± 8.51	<0.001
LVEDV, mL		122.00 (99.00, 150.00)	121.00 (96.00, 145.00)	126.00 (101.00, 155.00)	0.011
LVESV, mL		45.00 (32.00, 62.00)	41.00 (30.00, 57.00)	47.00 (35.00, 70.00)	<0.001
Angiography and procedure					
Intraoperative TIMI grade III, %		1087 (98.9)	546 (99.5)	541 (98.4)	0.082
Infarct-related artery, %					
	RCA	414 (37.7)	190 (34.6)	224 (40.7)	
	LAD	585 (53.2)	309 (56.3)	276 (50.2)	0.098
	LCX	100 (9.1)	50 (9.1)	50 (9.1)	
Average length of implanted stents, mm		24.35 ± 6.48	24.12 ± 6.48	24.58 ± 6.47	0.258
Average diameter of implanted stents, mm		3.22 ± 1.23	3.18 ± 0.86	3.25 ± 1.51	0.334
Gensini score		64.00 (42.00, 89.00)	64.00 (40.00, 87.00)	68.00 (44.00, 92.00)	0.009
Medication					
Aspirin, %		1076 (97.9)	541 (98.5)	535 (97.3)	0.141
P2Y12 inhibitor, %		1055 (96.0)	545 (99.3)	510 (92.7)	<0.001
Statins, %		1046 (95.2)	535 (97.4)	511 (92.9)	0.068
ACEI/ARB/ARNI, %		489 (44.5)	249 (45.4)	240 (43.6)	0.917
Beta-blocker, %		776 (70.6)	416 (75.8)	360 (65.5)	0.002

BMI, body mass index; SBP, systolic blood pressure; DBP, diastolic blood pressure; HR, heart rate; RBC, red blood cell; WBC, white blood cell; NEU, neutrophil; LYM, lymphocyte; MO, monocyte; PLT, platelet; CRP, C-reactive protein; ALT, alanine aminotransferase; AST, aspartate aminotransferase; STB, serum total bilirubin; CB, conjugated bilirubin; BUN, blood urea nitrogen; Cre, creatinine; UA, uric acid; eGFR, estimated glomerular filtration rate; TC, total cholesterol; TG, triglyceride; HDL-C, high-density lipoprotein cholesterol; LDL-C, low-density lipoprotein cholesterol; BAR, blood urea nitrogen-to-albumin ratio; LVEF, left ventricular ejection fraction; LVFS, left ventricular fractional shortening; LVEDV, left ventricular end diastolic volume; LVESV, left ventricular end systolic volume; TIMI, Thrombolysis in myocardial infarction; RCA, right coronary artery; LAD, left anterior descending artery; LCX, left circumflex artery; ACEI, angiotensin converting enzyme inhibitor; ARB, angiotensin receptor blocker; ARNI, angiotensin receptor-neprilysin inhibitor.

In addition, to provide complementary clinical insights, we also created Table 2 by grouping outcomes. We found that compared with the survivors group, the non-survivors group had older patients, more females, less smokers, and higher levels of white blood cell (WBC) count, neutrophil (NEU) count, MO, CRP, STB, CB, BUN, creatinine, UA, serum potassium, myoglobin, and BAR, as well as a higher Gensini score. Conversely, they had significantly lower BMI, DBP, RBC, HB, ALB, eGFR, TC, TG, LDL-C, and LVEF.

**Table 2.** **Baseline characteristics according to outcome**.

Variables		Totality population	Survivors	Non-survivors	*p* value
		n = 1099	n = 971	n = 128	
Basic characteristics					
Age, years		60.74 ± 12.07	59.51 ± 11.68	70.03 ± 10.97	<0.001
Sex, female, %		171 (15.6)	140 (14.4)	31 (24.2)	0.004
BMI, kg/m^2^		24.29 ± 3.13	24.36 ± 3.15	23.72 ± 2.95	0.032
SBP, mmHg		126.68 ± 23.59	126.85 ± 23.70	125.35 ± 22.77	0.499
DBP, mmHg		81.20 ± 16.12	81.60 ± 16.08	78.15 ± 16.15	0.023
HR, beat/minute		78.85 ± 17.07	78.80 ± 16.62	79.27 ± 20.21	0.798
Smoking, %		627 (57.1)	570 (58.7)	57 (44.5)	0.002
Medical history					
Hypertension, %		615 (56.0)	522 (53.8)	93 (72.7)	<0.001
Diabetes, %		346 (31.5)	296 (30.5)	50 (39.1)	0.050
Hyperlipidemia, %		350 (31.8)	330 (34.0)	20 (15.6)	<0.001
Atrial fibrillation, %		25 (2.3)	20 (2.1)	5 (3.9)	0.188
Heart failure, %		299 (27.2)	231 (23.8)	68 (53.1)	<0.001
Laboratory data					
RBC, 10^12^/L		4.91 ± 0.66	4.94 ± 0.67	4.69 ± 0.79	0.001
Hemoglobin, g/L		152.84 ± 23.01	153.82 ± 22.67	145.24 ± 24.31	<0.001
WBC, 10^9^/L		11.03 ± 4.59	10.93 ± 4.63	11.81 ± 4.20	0.044
NEU, 10^9^/L		8.50 ± 3.74	8.40 ± 3.69	9.25 ± 4.08	0.030
LYM, 10^9^/L		1.70 ± 0.83	1.71 ± 0.85	1.62 ± 0.71	0.242
MO, 10^9^/L		0.65 ± 0.35	0.63 ± 0.35	0.76 ± 0.39	0.001
PLT, 10^9^/L		225.32 ± 72.77	225.45 ± 71.59	224.30 ± 81.69	0.869
CRP, mg/L		3.38 (1.20, 12.80)	3.18 (1.09, 11.55)	6.25 (2.70, 38.11)	<0.001
Albumin, g/L		39.63 ± 4.48	39.89 ± 4.30	37.64 ± 5.24	<0.001
ALT, IU/L		43.00 (29.00, 63.00)	43.00 (30.00, 63.00)	39.55 (28.00, 66.75)	0.518
AST, IU/L		73.50 (32.00, 175.85)	73.50 (31.00, 173.00)	73.00 (37.25, 234.30)	0.187
STB, µmol/L		12.00 (8.80, 17.20)	12.00 (8.70, 16.90)	13.65 (9.43, 20.00)	0.016
CB, µmol/L		3.60 (2.50, 5.40)	3.50 (2.50, 5.30)	3.80 (2.83, 6.95)	0.007
BUN, mmol/L		5.60 (4.53, 6.90)	5.50 (4.40, 6.64)	6.77 (5.33, 9.35)	<0.001
Cre, µmol/L		89.70 (76.40, 103.80)	88.30 (75.10, 101.10)	103.10 (83.13, 137.20)	<0.001
UA, µmol/L		384.55 (317.75, 465.98)	377.10 (313.35, 456.65)	450.50 (351.70, 543.50)	<0.001
eGFR, mL/min/1.73 m^2^		72.28 (55.55, 90.95)	74.65 (58.35, 92.84)	50.02 (33.44, 71.54)	<0.001
Potassium, mmol/L		3.73 ± 0.54	3.71 ± 0.52	3.84 ± 0.63	0.030
Sodium, mmol/L		138.89 ± 3.73	138.95 ± 3.65	138.37 ± 4.25	0.144
TC, mmol/L		4.44 (3.70, 5.17)	4.47 (3.75, 5.22)	4.15 (3.38, 4.88)	0.002
TG, mmol/L		1.48 (1.04, 2.06)	1.50 (1.06, 2.11)	1.29 (0.93, 1.87)	0.004
HDL-C, mmol/L		1.04 (0.88, 1.22)	1.04 (0.88, 1.22)	1.06 (0.90, 1.21)	0.844
LDL-C, mmol/L		2.80 (2.19, 3.48)	2.84 (2.23, 3.53)	2.58 (1.92, 3.30)	0.004
Myoglobin, ng/mL		156.35 (58.45, 367.73)	141.71 (53.28, 353.50)	240.87 (106.37, 478.00)	<0.001
Cardiac Troponin I, ng/mL		2.37 (0.12, 15.25)	2.31 (0.11, 15.00)	3.65 (0.31, 18.01)	0.178
CK-MB, ng/mL		19.60 (3.58, 72.10)	20.30 (3.33, 73.36)	16.89 (6.00, 69.51)	0.660
BAR		3.91 (3.13, 5.03)	3.79 (3.04, 4.85)	5.08 (3.85, 6.97)	<0.001
Echocardiographic data					
LVEF, %		61.36 ± 11.55	62.02 ± 11.23	55.51 ± 12.68	<0.001
LVFS, %		34.01 ± 8.44	34.51 ± 8.27	29.63 ± 8.68	<0.001
LVEDV, mL		122.00 (99.00, 150.00)	121.00 (99.00, 147.00)	135.00 (103.00, 168.25)	0.006
LVESV, mL		45.00 (32.00, 62.00)	44.00 (32.00, 60.00)	58.00 (35.25, 77.25)	<0.001
Angiography and Procedure					
Intraoperative TIMI grade III, %		1087 (98.9)	964 (99.3)	123 (96.1)	0.001
Infarct-related artery, %					
	RCA	414 (37.7)	369 (38.0)	45 (35.2)	
	LAD	585 (53.2)	511 (52.6)	74 (57.8)	0.473
	LCX	100 (9.1)	91 (9.4)	9 (7.0)	
Average length of implanted stents, mm		24.35 ± 6.48	24.23 ± 6.40	25.34 ± 7.05	0.085
Average diameter of implanted stents, mm		3.22 ± 1.23	3.22 ± 1.18	3.21 ± 1.58	0.940
Gensini score		64.00 (42.00, 89.00)	64.00 (42.00, 88.00)	82.00 (46.00, 108.00)	<0.001
Medication					
Aspirin, %		1076 (97.9)	951 (97.9)	125 (97.7)	0.833
P2Y12 inhibitor, %		1055 (96.0)	933 (96.1)	122 (95.3)	0.675
Statins, %		1046 (95.2)	957 (98.6)	89 (69.5)	<0.001
ACEI/ARB/ARNI, %		489 (44.5)	454 (46.8)	35 (27.3)	0.019
Beta-blocker, %		776 (70.6)	716 (73.7)	60 (46.9)	0.001

### 3.2 Kaplan–Meier Analyses

**Fig. 2.**
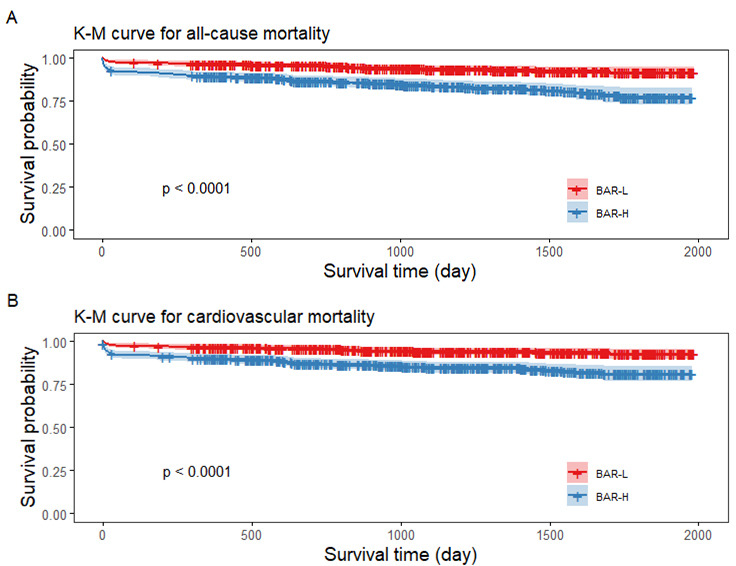
**Kaplan‒Meier curves for the cumulative rate of long-term all-cause mortality (A) and cardiovascular mortality (B) according to BAR**.

Kaplan–Meier survival analyses were conducted to evaluate associations between BAR levels and the risks of all-cause mortality and cardiovascular mortality. Collectively, elevated BAR levels were significantly linked to a higher risk of both outcomes (Fig. 2).

### 3.3 RCS Analyses

**Fig. 3.**
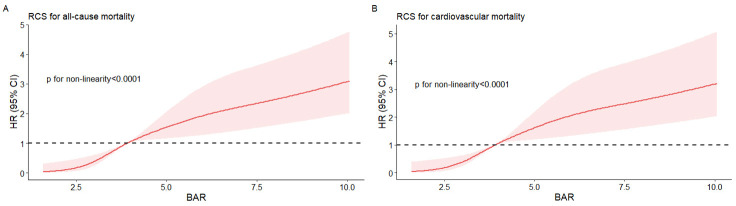
**Restricted cubic spline (RCS) for all-cause mortality (A) and cardiovascular mortality (B)**.

Restricted cubic spline (RCS) analyses were conducted to further explore the potential nonlinear relationship between BAR and the risks of all-cause mortality and cardiovascular mortality. The results indicated a nonlinear association between BAR and both outcomes (Fig. 3, *p* for nonlinear <0.0001).

### 3.4 Cox Regression Analyses

Univariate and multivariate Cox regression analyses were performed to further evaluate the relationship between the BAR and all-cause mortality and cardiovascular mortality (Table 3 and Table 4). The results demonstrated that higher BAR levels were significantly associated with an increased risk of both all-cause mortality and cardiovascular mortality. After adjustment for age, sex, BMI, hypertension, diabetes, smoking, Gensini score, intraoperative TIMI grade III, multivessel disease, LVEF, HB, eGFR, and LDL-C, the hazard ratio (HR) of BAR for all-cause mortality was 1.118 (95% CI: 1.049–1.193; *p* = 0.001). Similarly, after adjustment for age, sex, hypertension, diabetes, smoking, Gensini score, intraoperative TIMI grade III, multivessel disease, LVEF, WBC, HB, eGFR, and LDL-C, the HR for cardiovascular mortality was 1.107 (95% CI: 1.035–1.184; *p* = 0.003) for BAR. Collectively, these data demonstrate that BAR independently predicts both all-cause and cardiovascular mortality outcomes in STEMI patients treated with PPCI. In addition, as BAR is a composite indicator directly calculated from BUN and ALB, we constructed multivariable Cox regression models including BUN and albumin (Table 5) to evaluate their respective associations with outcomes. These models were then compared with models based on BAR.

**Table 3.** **Cox univariate analysis and multivariate analysis of all-cause mortality**.

Variables		Univariate analysis		Multivariate analysis	
		HR (95% CI)	*p* value	HR (95% CI)	*p* value
Age		1.081 (1.064–1.099)	<0.001	1.033 (1.007–1.059)	0.011
Sex					
	Male	reference		reference	
	Female	1.887 (1.259–2.829)	0.002	0.756 (0.446–1.283)	0.300
BMI		0.935 (0.882–0.992)	0.025	0.997 (0.927–1.071)	0.926
Hypertension		2.230 (1.505–3.302)	<0.001	1.567 (1.006–2.440)	0.047
Diabetes		1.488 (1.043–2.123)	0.028	1.661 (1.123–2.457)	0.011
Smoking		0.568 (0.401–0.805)	0.001	0.771 (0.484–1.229)	0.274
Gensini score		1.008 (1.005–1.012)	<0.001	1.008 (1.003–1.012)	0.001
Intraoperative TIMI grade III		0.187 (0.077–0.459)	<0.001	0.281 (0.100–0.787)	0.016
Multivessel disease		2.197 (1.152–4.190)	0.017	1.144 (0.516–2.536)	0.741
Myo		1.001 (1.000–1.001)	0.050		
cTnI		1.002 (0.996–1.008)	0.544		
CK-MB		1.000 (0.996–1.003)	0.923		
LVEF		0.971 (0.957–0.985)	<0.001	0.982 (0.966–0.998)	0.026
WBC		1.020 (0.999–1.042)	0.063		
HB		0.985 (0.978–0.992)	<0.001	1.001 (0.992–1.010)	0.783
PLT		1.000 (0.997–1.002)	0.793		
eGFR		0.961 (0.953–0.968)	<0.001	0.977 (0.964–0.991)	0.001
TG		0.851 (0.704–1.029)	0.095		
HDL-C		0.967 (0.780–1.199)	0.761		
LDL-C		0.770 (0.637–0.931)	0.007	0.855 (0.692–1.057)	0.147
BAR		1.159 (1.128–1.190)	<0.001	1.118 (1.049–1.193)	0.001

HR, hazard ratio; CI, confidence interval.

**Table 4.** **Cox univariate analysis and multivariate analysis of cardiovascular mortality**.

Variables		Univariate analysis		Multivariate analysis	
		HR (95% CI)	*p* value	HR (95% CI)	*p* value
Age		1.072 (1.054–1.091)	<0.001	1.026 (1.000–1.052)	0.050
Sex					
	Male	reference		reference	
	Female	1.989 (1.305–3.033)	0.001	0.863 (0.498–1.496)	0.600
BMI		0.941 (0.885–1.001)	0.053		
Hypertension		2.257 (1.486–3.427)	<0.001	1.658 (1.046–2.627)	0.031
Diabetes		1.557 (1.071–2.264)	0.028	1.739 (1.156–2.618)	0.008
Smoking		0.599 (0.414–0.866)	0.006	0.805 (0.492–1.319)	0.390
Gensini score		1.010 (1.006–1.013)	<0.001	1.009 (1.004–1.013)	<0.001
Intraoperative TIMI grade III		0.170 (0.069–0.418)	<0.001	0.271 (0.096–0.765)	0.014
Multivessel disease		2.450 (1.194–5.027)	0.015	1.412 (0.574–3.472)	0.453
Myo		1.001 (1.000–1.001)	0.052		
cTnI		1.000 (0.991–1.008)	0.918		
CK-MB		1.000 (0.997–1.004)	0.878		
LVEF		0.966 (0.952–0.981)	<0.001	0.978 (0.962–0.995)	0.013
WBC		1.022 (1.001–1.044)	0.040	1.028 (1.003–1.054)	0.026
HB		0.984 (0.977–0.992)	<0.001	1.000 (0.991–1.010)	0.985
PLT		0.999 (0.996–1.002)	0.438		
eGFR		0.962 (0.955–0.970)	<0.001	0.978 (0.966–0.991)	0.001
TG		0.867 (0.714–1.054)	0.152		
HDL-C		0.961 (0.710–1.300)	0.794		
LDL-C		0.768 (0.628–0.940)	0.011	0.871 (0.700–1.084)	0.216
BAR		1.154 (1.121–1.188)	<0.001	1.107 (1.035–1.184)	0.003

**Table 5.** **Cox analysis of BUN, ALB, and BAR**.

Variables	All-cause mortality		Cardiovascular mortality	
	HR (95% CI)	*p* value	HR (95% CI)	*p* value
BUN	1.080 (1.023–1.139)	0.005	1.069 (1.009–1.133)	0.023
ALB	0.954 (0.912–0.998)	0.042	0.948 (0.903–0.994)	0.029
BAR	1.118 (1.049–1.193)	0.001	1.107 (1.035–1.184)	0.003

All-cause mortality: Adjust for Age, Sex, BMI, Hypertension, Diabetes, Smoking, Gensini score, Intraoperative TIMI grade III, Multivessel Disease, LVEF, HB, eGFR, and LDL-C. Cardiovascular mortality: Adjust for Age, Sex, Hypertension, Diabetes, Smoking, Gensini score, Intraoperative TIMI grade III, Multivessel Disease, LVEF, WBC, HB, eGFR, and LDL-C.

### 3.5 Subgroup Analyses

**Fig. 4.**
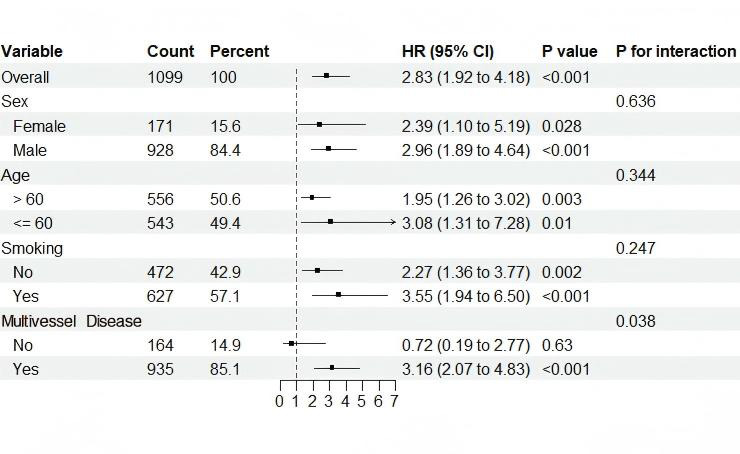
**The relationship between BAR and all-cause mortality in different subgroups of populations**.

**Fig. 5.**
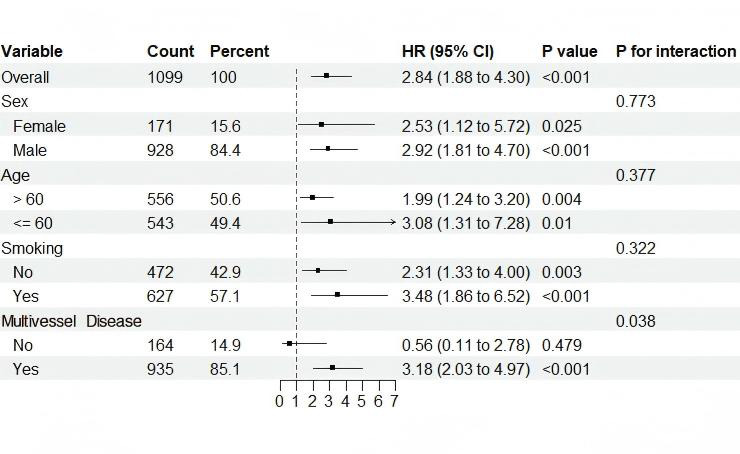
**The relationship between BAR and cardiovascular mortality in different subgroups of populations**.

Subgroup analyses were conducted to examine how BAR correlates with all-cause mortality and cardiovascular mortality across distinct patient subgroups, stratified by age, sex, smoking status, and the presence of multivessel disease. The results showed that higher BAR levels were significantly linked to increased risks of both mortality endpoints in subgroups defined by age, sex, or smoking status. In contrast, no significant association emerged between BAR and either mortality outcome in patients without multivessel disease (Figs. 4,5).

### 3.6 Time-Dependent ROC Analyses

**Fig. 6.**
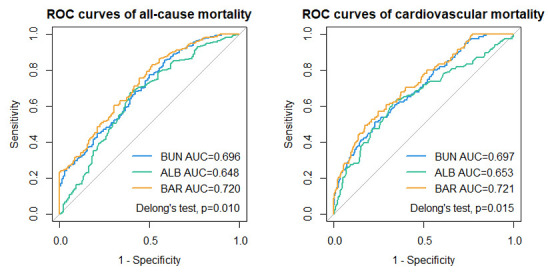
**Time-dependent receiver operating characteristic (ROC) curves of BUN, ALB, and BAR for all-cause mortality and cardiovascular mortality**. AUC, area under the curve; CI, confidence interval.

Receiver operating characteristic (ROC) curve analyses were conducted to assess and compare the predictive efficacy of BUN, ALB, and BAR for all-cause mortality and cardiovascular mortality. As illustrated in Fig. 6 and Table 6, the areas under the ROC curve (AUC) for all-cause mortality were 0.696 for BUN, 0.648 for ALB, and 0.720 for BAR; for cardiovascular mortality, the corresponding AUC values were 0.697 for BUN, 0.653 for ALB, and 0.721 for BAR. BAR demonstrated significantly superior predictive performance compared to both BUN and ALB for either endpoint (DeLong’s test *p* < 0.05).

**Table 6.** **The ROC curve analysis to determine the optimal cut-off levels for BUN, ALB, and BAR in predicting all-cause mortality and cardiovascular mortality**.

		Youden index J	Cut-off	Sensitivity	Specificity	AUC	95% CI	*p* value
BUN	All-cause mortality	0.2745	6.85	0.5000	0.7745	0.696	0.668–0.723	<0.001
	Cardiovascular mortality	0.2803	6.85	0.5088	0.7716	0.697	0.669–0.724	<0.001
ALB	All-cause mortality	0.2819	38.40	0.6094	0.6725	0.648	0.619–0.676	<0.001
	Cardiovascular mortality	0.2929	38.40	0.6228	0.6701	0.653	0.624–0.681	<0.001
BAR	All-cause mortality	0.3134	5.27	0.4844	0.8290	0.720	0.693–0.747	<0.001
	Cardiovascular mortality	0.3214	4.86	0.5702	0.7513	0.721	0.694–0.747	<0.001

## 4. Discussion

We retrospectively analyzed 1099 STEMI patients who underwent PPCI to investigate the relationship between BAR and long-term outcomes. Kaplan-Meier curves confirmed elevated BAR levels correlated with an increased risk of mortality, while restricted cubic spline (RCS) analysis uncovered a significant nonlinear relationship—suggesting a threshold effect wherein BAR’s prognostic impact intensifies beyond a critical value. Multivariate Cox regression further validated BAR’s independence as a risk marker after adjusting for confounders. The predicted value of the BAR index is more pronounced in patients with multivessel disease, likely because multivessel disease causes more severe myocardial ischemia—amplifying the impact of BAR on mortality. In the presence of non-multivessel disease, ischemia is less extensive, so the effect of BAR is masked by fewer competing risk factors. This suggests BAR is most useful for risk stratification in high-risk STEMI patients. ROC curve analysis showed that BAR had significantly better predictive performance than BUN and ALB in predicting all-cause mortality and cardiovascular mortality. Collectively, these findings suggest that BAR serves as a robust prognostic indicator for STEMI patients after PPCI. The exact reasons and potential pathophysiological mechanisms underlying the association between BAR and long-term adverse outcomes after PPCI in STEMI patients are still unclear. Therefore, we can only analyze the possible mechanisms by focusing on the main components that make up BAR, namely BUN and ALB.

BUN serves as a biomarker of renal function, and its serum concentration not only reflects glomerular filtration but also has a close relationship with tubular reabsorption/excretion. Another renal function marker, estimated glomerular filtration rate (eGFR), has also been linked to AMI prognosis. However, eGFR calculated solely based on creatinine may not accurately reflect renal function [19]. Elevated BUN levels are also associated with activation of the renin angiotensin aldosterone system and sympathetic nervous system, which may be caused by renal hypoperfusion due to low blood volume, renal vascular disease, or reduced cardiac output [20]. During the acute phase of myocardial infarction, low cardiac output, hypotension, and renal hypoperfusion are common. The reabsorption of sodium and water is regulated by antidiuretic hormone under the influence of angiotensin-II, while the reabsorption of urea occurs passively together with the reabsorption of sodium and water [21]. In the context of a STEMI, the increase of BUN often precedes the increase of serum creatinine, and its significance goes far beyond simple renal dysfunction. Therefore, BUN could serve as an encompassing marker by reflecting impaired cardiorenal function and neurohormonal activation. Among acute decompensated heart failure (ADHF) patients, an increased BUN level has been demonstrated to be a stronger marker of mortality than creatinine [22]. A prospective cohort study by Horiuchi et al. [23] found that elevated BUN levels were tied to higher in-hospital mortality and major adverse cardiovascular events (MACE) in AMI patients, and independently predicted in-hospital mortality. In summary, since BUN effectively mirrors acute hemodynamic and neurohumoral shifts, it has significant prognostic value in STEMI patients.

Albumin (ALB), the most abundant plasma protein, performs key physiological roles—maintaining colloid osmotic pressure and transporting diverse substrates. It is well-established that ALB mirrors systemic inflammation: as a hepatically synthesized protein, it is reduced in both acute and chronic inflammatory states due to decreased synthesis and increased catabolism driven by inflammatory responses. Given that atherosclerosis is recognized as a chronic inflammatory disease, inflammation critically drives its progression and destabilization—processes central to cardiovascular pathogenesis [24]. The systemic inflammatory response in STEMI patients may be related to myocardial injury, repair, and remodeling. As part of the pro-inflammatory response that begins with myocardial injury, large amounts of cells and molecules are released into circulation. In infarcted myocardium, activated signals trigger Toll-like receptor signaling. Complement activation and reactive oxygen species production induce upregulation of cytokines and chemokines. White blood cells are recruited to the infarcted area, where they engulf and clear dead cells along with matrix debris, while simultaneously preparing the tissue for scar formation [25]. This inflammatory milieu is likely responsible for the prognostic value of ALB. Research has shown that decreased serum albumin levels upon admission are an independent predictor of long-term mortality in patients with AMI [26]. Hypoalbuminemia has been identified as an independent risk for slow flow after PCI and mortality in patients with AMI [27,28]. Similarly, it has been found that hypoalbuminemia is associated with poorer clinical outcomes in heart failure, stroke, and cancer [29,30,31].

Malnutrition may also contribute to the link between low serum ALB levels and unfavorable clinical outcomes. Nutritional status is a key determinant of prognosis across various diseases, and malnutrition—marked by depleted protein and energy stores—compromises immune function. Prior studies have linked malnutrition to an elevated risk of cardiovascular events in patients with heart failure [32]. Kanda et al. [33] identified malnutrition as an independent predictor of all-cause mortality in AMI patients who underwent PCI. In addition, albumin may also be associated with promoting platelet aggregation and thrombus formation [34]. Stenvinkel et al. [35] found that compared with well-nourished dialysis patients and patients with normal CRP levels, malnourished dialysis patients and patients with elevated CRP levels had significantly greater carotid intima media thickness, indicating that inflammation and nutrition may be key factors in the development of atherosclerosis.

At present, BAR, a novel combined indicator composed of BUN and ALB, has been used as a new biomarker to assess the prognosis of various diseases and is an important prognostic factor for mortality in several diseases, such as heart failure, pulmonary embolism, and lung cancer [12,13,14]. These findings suggested that the BAR index can serve as a valuable tool for risk stratification. As a composite index integrating BUN and ALB, BAR offers a holistic reflection of physiological reserve, capturing multidimensional factors such as malnutrition, dehydration, and renal function that may refine disease severity assessment and yield superior predictive value versus single markers. Zhang et al. [20] suggest that elevated BAR independently predicts left ventricular aneurysm development in STEMI patients. Several studies have also shown BAR to be an independent determinant of mortality risk among AMI patients [15,16]. In the study by Balcik et al. [16], admission BAR in STEMI patients was predictive of short-term mortality—each unit rise in BAR corresponded to a 1.25-fold increase in the risk of mortality. However, the association between BAR and long-term outcomes in PPCI-treated STEMI patients remains incompletely understood. Thus, our study sought to investigate this relationship.

In our study, we first assessed BAR’s ability to predict long-term outcomes in post-PPCI STEMI patients, finding that elevated BAR independently correlated with increased risks of all-cause and cardiovascular mortality. The prognostic value of BAR was attributable to its components, BUN and ALB. Therefore, it can be reasonably inferred that BAR is a reliable prognostic indicator for postoperative PPCI in STEMI patients. Elevated BAR values in patients may indicate overactivation of the RAAS system, inadequate renal perfusion, systemic inflammatory response, and/or malnutrition, which can lead to a poorer prognosis. Physicians can use BAR to stratify the risk of STEMI patients and provide early intervention and personalized treatment to improve their prognosis.

### Limitations

First, this was a single-center retrospective analysis. Although we conducted multivariate analysis to adjust for potential risk factors, residual confounding factors may affect clinical outcomes. Second, our research findings have not been validated in different hospitals or patient populations and may include specific risk conditions based on epidemiological or geographic factors. Third, BAR was only calculated at admission, and the prognostic effects of its dynamic changes are not yet clear. In the future, research on continuous BAR measurements is needed to explore whether dynamic changes can enhance its prognostic value.

## 5. Conclusions

BAR is independently associated with long-term survival outcomes in STEMI patients undergoing PPCI and exhibits strong predictive capability, positioning it as a valuable tool for risk stratification. Clinicians can leverage this index to identify high-risk patients, enabling timely early intervention and personalized care to improve outcomes.

## Data Availability

The datasets that were used and analyzed during the current study are available from the corresponding author upon reasonable request.
